# The postpartum uterus reveals compartment-specific remodeling processes with distinct immune signatures

**DOI:** 10.3389/fimmu.2026.1824792

**Published:** 2026-06-10

**Authors:** Antonia Waldmann, Yvonne Natascha Susanne Weiß, Lilja Hardardottir, Maurice Kappelmeyer, Maria Victoria Bazzano, Wenqin Shi, Angela Köninger, Maria Emilia Solano

**Affiliations:** 1 Laboratory for Tranlational Perinatology, Department of Gynecology and Obstetrics, University of Regensburg, Regensburg, Germany; 2 Clinic St. Hedwig of the Order of St. John, Department of Gynecology and Obstetrics, University of Regensburg, Regensburg, Germany

**Keywords:** macrophages, postpartum, T cells, tissue regeneration, tissue remodeling, uterus

## Abstract

**Introduction:**

The endometrium exhibits the ability to regenerate without scarring after menstruation or parturition. Postpartum uterine repair occurs in a unique environment, shaped by peripartum immune responses. However, the mechanisms leading to healing or excessive fibrosis remain poorly understood. This study aimed to evaluate the drastic postpartum changes and the involvement of the immune milieu in the process of full wound regeneration.

**Methods:**

Publicly available scRNA-seq data of human myometrium from non-pregnant or postpartum day 7 individuals were analyzed. Allogenically mated and virgin C57/BL6 female mice served as models for human postpartum healing. Uterine tissues were analyzed by flow cytometry, immunohistochemistry, immunofluorescence, and quantitative real-time polymerase chain reaction.

**Results:**

Human myometrial scRNA-seq revealed a sustained upregulation of immune responses in a context of degrowth of muscle components by postpartum day 7. As shown in mice, the size of the myometrium rapidly retracts. Particularly, the gradual resorption of the main site of leukocyte infiltration during pregnancy and postpartum, the mesometrial triangle, grants restoration of myometrial layers, dispersed during pregnancy to accommodate the leucocyte aggregates and growing conceptus. This region, encircling the lesions left by placental detachment, abundantly recruits macrophages that remain CD206^neg^. This inflammatory reaction coincides with elevated levels of *Tnf*, *Il2*, and *Ifng*, neovascularization, and cell death, and is balanced by increased *Il10* levels. CD8^+^ T cells also seeded the mesometrial triangle, along with myofibroblasts surrounding the placental detachment clot until healing. This immune response was confined to the mesometrial area by a collagen-rich capsule. In turn, neighboring myometrial macrophages, largely expressing CD206, expanded during early remodeling to retract at homeostasis.

**Conclusion:**

In healthy postpartum uterus tissue, we identified the myometrium, endometrium, and mesometrial triangle as distinct anatomical compartments undergoing remodeling, each with unique yet interconnected processes. Within these, the distribution of F4/80^+^CD206^+^ and F4/80^+^CD206^neg^ macrophage populations corresponded to different levels of tissue disruption. Inflammation, highly restricted by a collagen capsule and enhanced IL10, resolved to endorse myometrium integrity. Whether these mechanisms safeguard the myometrium from excessive immune responses, often linked to fibrosis, requires further empirical investigation.

## 1 Introduction

In most mammals, the uterine wall consists of three principal layers: The endometrium as the innermost layer, the myometrium in the middle, and the perimetrium (serosa) as the outermost layer. Together, these tunicae enable cyclic remodeling, contractility, and the support of pregnancy. The endometrium is known for its capability of scarless healing during the menstrual cycle or after parturition. However, so far, it is not fully understood which processes enable this healing capacity. Several theories have been proposed to explain it, these include (i) the proliferation of epithelial cells of endometrial glands in the basal layer, (ii) reprogramming of mesenchymal cells to epithelial cells, (iii) differentiation of endometrial stem cells into stromal epithelial cells (1).

In contrast, wound healing in tissues such as the skin is better studied and involves overlapping phases (2). First, the hemostasis and inflammation phase results in the formation of a temporary extracellular matrix and the recruitment of innate immune cells, such as neutrophils, monocytes, and macrophages, to the injury site (3). Next, the proliferation phase is characterized by re-epithelialization, angiogenesis, and the formation of granulation tissue, rich in collagen III (4). Granulation tissue forms in parallel with angiogenesis and consists of collagen III, fibroblasts, and newly formed blood vessels. Fibroblasts play a central role in this process and are activated by macrophage-derived mediators such as TNF-α, IL-6, and TGF-β, thereby promoting re-epithelialization and matrix deposition (5). In the final remodeling phase, collagen I replaces collagen III, angiogenesis subsides, and myofibroblasts contract the wound, typically resulting in scar formation (6). During healing, intense inflammation can lead to excessive angiogenesis and sustained leucocyte infiltration, perpetuating an inflammatory loop (4, 7). In fact, scarless tissues such as fetal skin or oral mucosa exhibit reduced inflammatory and angiogenic responses (8).

In the uterus, wound healing upon natural delivery or cesarean section occurs within a unique immune environment. Indeed, maternal immune tolerance to fetal antigens (9, 10) tilts to an inflammatory response during labor and parturition (11). In labor, inflammatory mediators such as IL-6 and TNF-α in the decidua, myometrium, or amniotic fluid (12, 13), trigger neutrophil and monocyte infiltration (12), which may affect postpartum uterine tissue healing. In mice, an increased contractile tone after birth supports clot resorption, hemostasis, and epithelial repair (14). However, the mechanisms underlying labor-related changes in uterine healing remain largely unknown.

Experimental evidence indicates that macrophages, which are chiefly involved in both wound fibrosis and healing (15), peak in the early postpartum phase (14). Inflammatory macrophages can drive fibrosis by secreting (i) enzymes such as matrix metalloproteinases that remodel the extracellular matrix (ECM), (ii) TGF-β1, and mediators that support fibroblast differentiation into myofibroblasts and thus ECM production (16, 17) and (iii) inflammatory cytokines (3, 15). Conversely, alternatively activated macrophages are generally acknowledged to aid healing by producing IL-10 and other immunoregulatory and anti-fibrotic mediators (18). Notably, macrophage polarization spans a spectrum that can shift dynamically in response to microenvironmental signals, including cytokines and cell-to-cell interactions (19).

Because of its ability to promote an inflammatory environment, the adaptive immune system was initially regarded as pro-fibrotic. Accordingly, the absence of T cells at the lesion has been associated with increased wound closure (20). More recently, a fine-tuned role of T cells in tissue healing has been recognized. The recruitment of specific T cell subsets within the first 24 h of wounding reduced scar building (21), and CD8^+^ T cell-derived IFN-γ limited tissue damage during infections (22).

Based on this evidence, this study sought to describe scarless physiological tissue regeneration in the postpartum uterus. We aimed to characterize postpartum healing by focusing on the most affected pathways identified in human myometrial samples and linking them to processes in uterine anatomy, vascular and lymphatic remodeling, and tissue turnover, as well as to dynamic changes in resident immune cell populations in mouse postpartum samples as an experimental model.

## 2 Material and methods

### 2.1 Bioinformatic analyses of human scRNA sequencing data

The publicly available dataset from Ulrich et al. (GSE260658) (23), including five non-pregnant myometrium samples and one 7-day postpartum sample, was analyzed with Seurat (version 5.3.0) (24). No information about the mode of delivery was given. Integration was facilitated with Seurat’s RPCA method. Differential gene expression (DEG) testing was performed with MAST (25), adjusted for cell type, to account for differences in cell type abundances between conditions. Genes were considered significantly differentially expressed if their adjusted p-values were below 0.05 and their absolute log2FC was above 0.25. Gene set enrichment analysis was performed using the Gene Ontology database with clusterProfiler (version 4.10.1) (26). The number of DEGs per cell type was calculated using the Wilcoxon Rank-Sum test.

### 2.2 Animal experiments and murine tissue collection

Female C57BL/6J virgin mice and male BALB/c mice (Charles River Laboratories) were housed under controlled conditions (12-hour light/dark cycle, ad libitum access to food and water) in accordance with institutional ethical guidelines. Vaginal smears from virgin mice were stained with hematoxylin and eosin to determine the stage of the estrous cycle (27). Timed pregnancies were conducted by co-housing female and male mice, with the detection of a vaginal plug defining gestation day 0.5 (GD0.5). The day of natural delivery was designated as postpartum day 1 (PPD1). All experiments were performed in accordance with the animal ethics approval given by the State Authority of Hamburg (N046/2020) and State Authority of Regensburg (RUF-55.2.2-2532-2–1488 and -2081). Tissues were harvested during pregnancy (GD6.5, GD18.5), postpartum (PPD2, PPD4, PPD21, PPD42), and virgin controls in diestrous and estrous stages. For pregnant samples, the fetus was removed from the uterine horns, and the remaining implantation site was collected, and for postpartum samples the former implantation sites were dissected.

### 2.3 Masson Goldner trichrome staining

Paraffin-embedded tissue sections (5 µm) were deparaffined and incubated with hematoxylin solution followed by immersion in ponceau, phosphotungstic acid, and light green solutions. Staining was enhanced using 1% glacial acetic acid between individual steps. Cytoplasm appeared in red, erythrocytes orange, collagen-rich connective tissue in blue.

### 2.4 Immunohistochemical analysis of αSMA, CD31, F4/80, CD206, CD8, collagen I

Sections were subjected to heat-mediated antigen retrieval (98 °C, 5 min) and quenching of endogenous peroxidase activity by 3% H_2_O_2_ (5 min). Sections were blocked for 1 h at room temperature and incubated with primary antibodies overnight at 4 °C, except for collagen, with 1.5 h primary antibody incubation. Next, followed the incubation with the respective secondary antibody (1 h) and HRP-conjugated streptavidin or alkaline phosphatase reagent (APC-AP kit), according to manufacturer’s instruction. Sections were counterstained with hematoxylin. Slides were scanned using the Scanner P1000 (3DHistech) and analyzed with Slideviewer 2.7.0, Fiji 2.16.0 (28) and QuPath 0.5.1 (29). Antibodies and dilutions are listed in **Supplementary Table 1**.

### 2.5 TUNEL Immunohistochemical analysis

Apoptosis was detected in uterine sections using the TACS^®^ 2 TdT-DAB *In Situ* Apoptosis Detection Kit according to the manufacturer’s instructions. Sections were subsequently counterstained with hematoxylin.

### 2.6 Immunofluorescence

Upon hydration and blocking, sections were incubated with the respective primary antibodies for 1h at room temperature, followed by fluorophore-conjugated secondary antibodies (1h) (**Supplementary Table 1**). Nuclei were counterstained with Hoechst 33342 (5 min). Slides were analyzed with Fluoview FV3000 Laser Scanning Microscope (Olympus).

### 2.7 Flow cytometry

Uterine samples were cut and digested in Accutase for 30 minutes at 37 °C. The resulting cell suspension was filtered, centrifuged, and residual red blood cells were removed using RBC lysis buffer. After washing with DPBS, Fc-receptors were blocked and cells stained with a viability stain and antibody mixture as shown in **Supplementary Table 1**. Subsequently, cells were fixed and permeabilized for intracellular antibodies. Samples were measured by the BD LSRFortessa Cell Analyzer and analyzed with FlowJo (v10.10.0).

### 2.8 RNA isolation, cDNA synthesis, and quantitative PCR

Mouse tissue samples were collected from virgin, pregnant, and postpartum uteri and immersed in RNAlater. RNA was isolated using RNeasy Plus Universal Mini Kit and reverse transcribed to cDNA using High-Capacity cDNA Reverse Transcription Kit according to manufacturer’s instructions. Efficiency was calculated based on standard curves using 500 ng to 1.9 ng cDNA for each target gene. Gene expression was quantified by qPCR with TaqMan^®^ primers (**Supplementary Table 1**) and Universal PCR Master Mix. Samples were normalized to the established reference gene Rpl13a (30).

### 2.9 Statistical analysis

Statistical analyses and design of graphs of experimental data were performed with GraphPad Prism version 10. After testing for normality with a Kolmogorov-Smirnov test, datasets were analyzed by Kruskal-Wallis test and Dunn’s correction for multiple testing with p<0.05 considered as statistically significant. Data is presented as the mean ± standard error of the mean.

## 3 Results

### 3.1 Upregulation of immune-responsive pathways and downregulation of muscle development in the postpartum uterus

**Figure 1:**
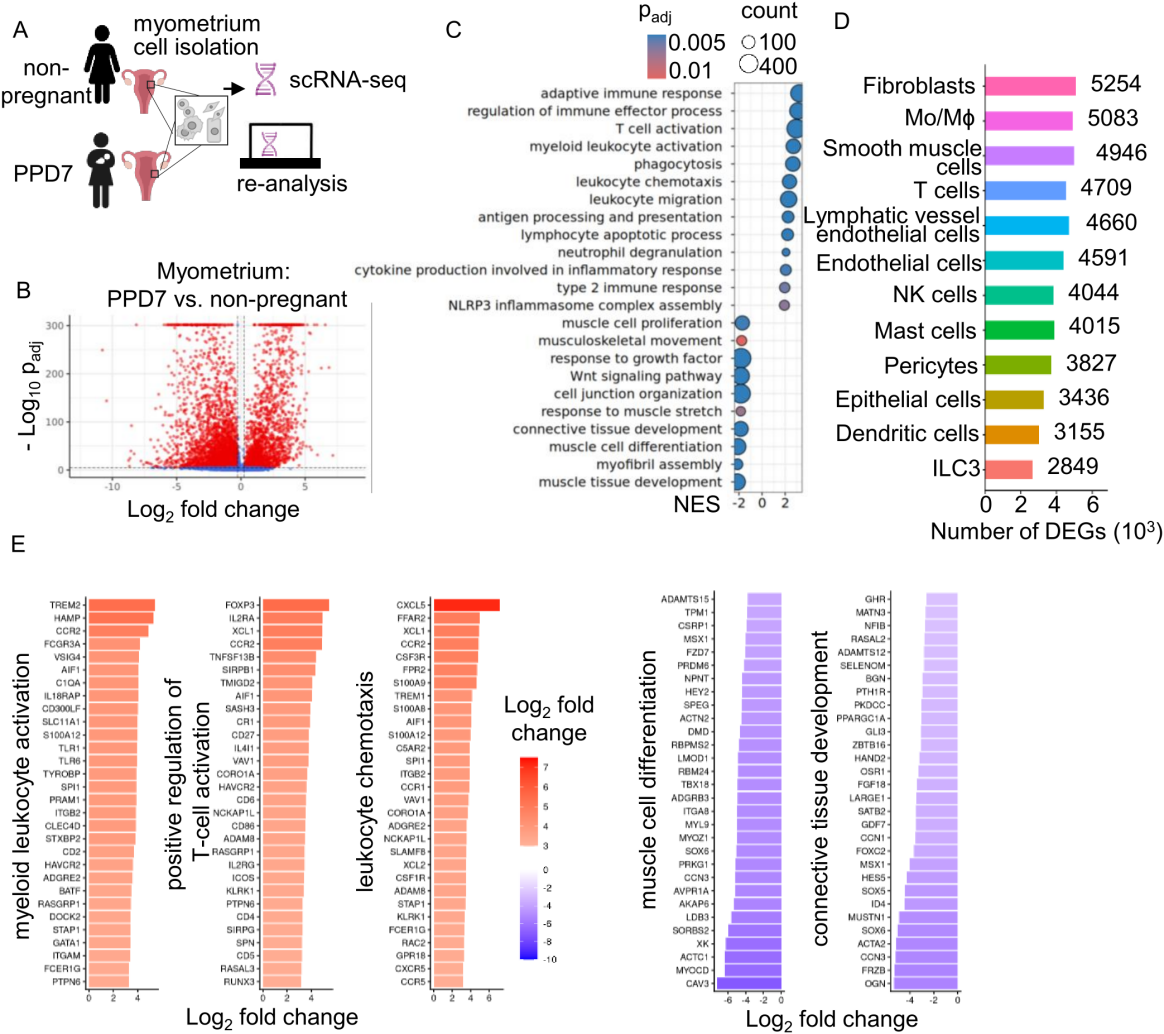
scRNA-seq data reveal exacerbated immune responses and signs of muscle involution in the PPD7 human uterus. **(A)** Sample collection and scRNA-seq by Illumina NovaSeq6000 of the myometrium from human PPD7 and non-pregnant uterus. Data set: GSE260658 (Ulrich et al.). **(B)** Volcano plot of significantly up- and downregulated DEGs in PPD7 uterus. **(C)** Gene Ontology (GO) pathways up- and downregulated in the PPD7 uterus. NES: normalized enrichment score. **(D)** Number of DEGs in common cell subsets on PPD7 myometrium. **(E)** Top 30 up- (top) or downregulated (bottom) genes in relevant pathways. DEG: differentially expressed genes, Mo/Mϕ: monocytes/macrophages, ILC: innate lymphoid cells, NK: natural killer.

We first aimed to establish a hierarchy of processes contributing to the substantial anatomical changes associated with postpartum regeneration. Despite access to human postpartum uterine tissue is limited, a rare single-cell RNA sequencing (scRNA-seq) dataset of healthy myometrial samples from premenopausal non-pregnant and postpartum day (PPD) 7 women was publicly available (23) and here re-analyzed (**Figure 1A**). The extent of structural remodeling was reflected by the substantial number of differentially expressed genes (DEGs) that were up- or downregulated in PPD7 compared to non-pregnant myometrium, even when controlled for cell type (**Figure 1B**). Gene Ontology (GO) analysis revealed that immune pathways were the most upregulated in the postpartum uterus, including the regulation of immune effector processes, cytokine production involved in inflammatory response, and leukocyte activation. In contrast, pathways associated with muscle development, such as muscle differentiation, myofibril assembly and muscle tissue development, were downregulated (**Figure 1C**). Examining specific cell types for DEGs from PPD7 versus non-pregnant myometrium in the scRNA-seq dataset indicated that most DEGs occurred in fibroblasts, monocytes/macrophages, smooth muscle cells, T cells and lymphatic endothelial cells (**Figure 1D**). A deeper evaluation revealed upregulation of *TREM2, HAMP, CCR2* and *FCGR3A* among genes involved in myeloid leukocyte activation, of *FOXP3* and *IL2RA* within positive regulation of T-cell activation, and of *CXCL5* and *FFAR2* among genes associated with leukocyte chemotaxis. Conversely, genes downregulated in muscle cell differentiation included *ACTC1, MYOCD* and *CAV3*, while within connective tissue development, genes *ACTA2, CCN3, FRZB*, and *OGN* were reduced (**Figure 1E**).

Hence, muscle and ECM regression, along with enhanced immune responses, appear central to postpartum healing in women. Given the unavailability of human postpartum uterine samples to confirm and further investigate these dynamic processes, we next investigated postpartum healing upon allogeneic mouse pregnancies. Similar to women, mice develop hemochorial placentas and substantial immune modulation that tilts towards inflammation at labor, providing a comparable scenario to investigate uterine healing.

### 3.2 Structural remodeling at the former implantation site involves re-epithelialization and resorption of a detachment clot

**Figure 2:**
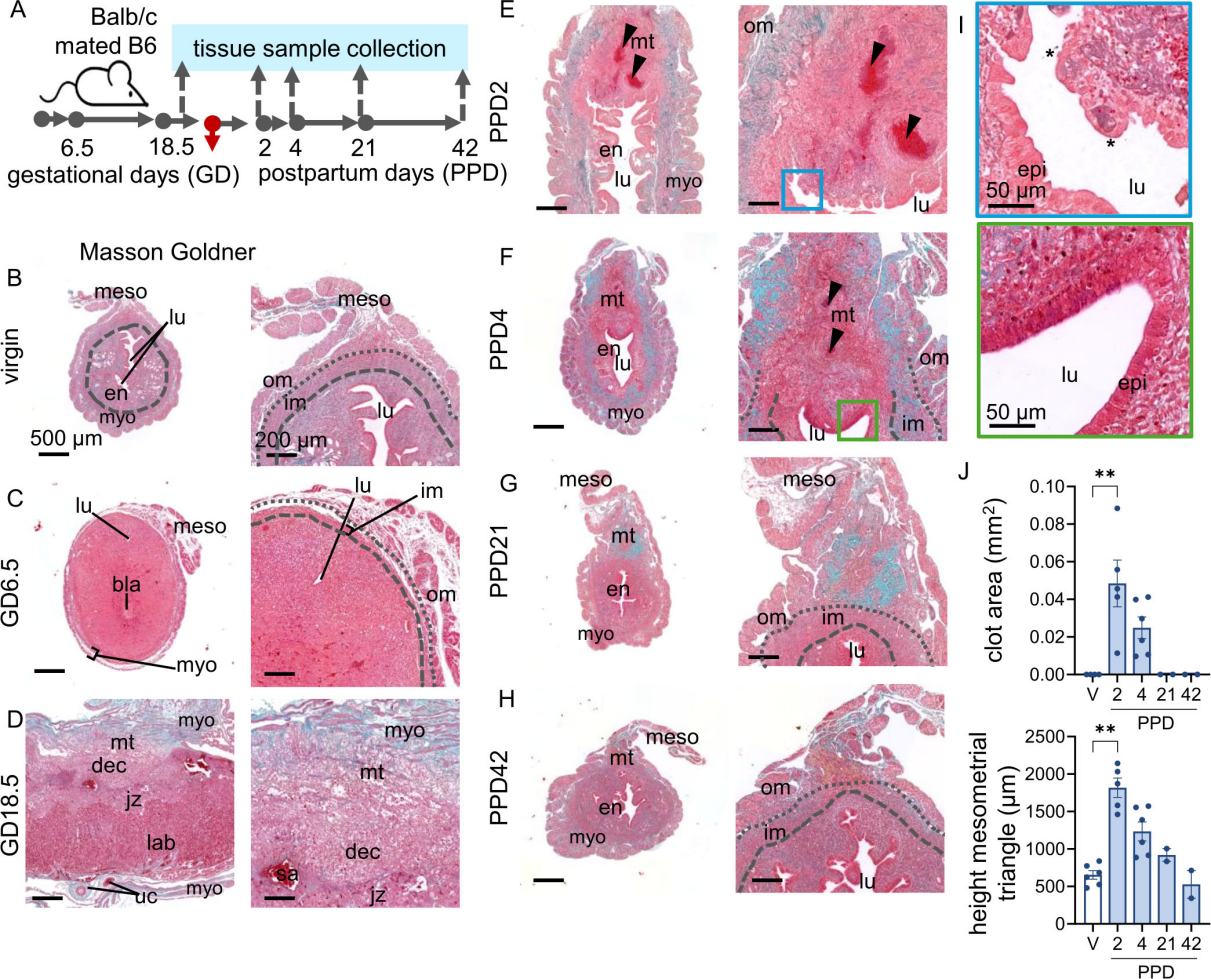
Impact of pregnancy on endometrial tissue in the postpartum uterus. **(A)** Mouse model implemented for the investigation of postpartum uterine regeneration. C57BL/6J females were mated with Balb/c males. The day of vaginal plug was defined as gestational day (GD)0.5. The day of delivery is defined as postpartum (PPD) 1. Uteri collected from virgin (V) mice on GD6.5, 18.5, PPD2, 4, 21, and 42. **(B-H)** Representative microphotographs of Masson Goldner Trichrome-stained uteri tissue sections. lu, lumen; im, inner myometrium; om, outer myometrium; myo, myometrium; en, endometrium; gl, gland; dec, decidua (basalis); jz, junctional zone; lab, labyrinth; sa, spiral artery; ac, amniotic cavity; uc, umbilical cord; epi, epithelium. Filled arrowheads: hemorrhagic clot. **(I)** Magnification of E (top, blue) and F (bottom, green) to show luminal epithelium. **(J)** Quantification of the clot area (top) and the height of the mesometrial triangle. (bottom) of uteri. Mean with SEM, *p < 0.05, **p < 0.01, Kruskal-Wallis test.

To investigate the pathways affected in the human postpartum uterus, namely muscle and ECM regression, and enhanced immune responses, allogenic mouse pregnancies were used as a model that mimics placental and immune features observed in women. Uterine samples were harvested before and after natural delivery, and from virgins as a control (**Figure 2A**). Throughout the study, analyses distinguished between two uterine regions: the mesometrial region, defined by the attachment to the mesometrium, a major component of the broad ligament, and comprising the placental attachment site, and the antimesometrial region on the opposite uterine wall. A progressive expansion of the uterine diameter over pregnancy was observed, resulting in mechanical stretching of the myometrial layers. Following natural parturition, a sharp reduction of uterine size was apparent on PPD2 and PPD4, additionally the inner myometrial layer was disrupted. By PPD42, uterine dimensions and myometrial layers were comparable to virgin controls (**Figures 2B–H**). This size decrease was also reflected in the extensive remodeling of the former decidua. The mesometrial triangle, the upper mesometrial portion of the murine uterus, adjacent to the decidua, which expands during pregnancy to accommodate the mesometrial lymphoid aggregate of pregnancy (MLAp) (31, 32), gradually reduced through the postpartum phase (**Figures 2B, E–H, J**). Furthermore, hemorrhagic clots formed at the site of placental detachment were most prominent on PPD2 within the mesometrial triangle (**Figures 2E, F**). These clots already showed reduced area by PPD4 (**Figure 2J**) and were entirely resorbed by PPD21 (**Figures 2H, J**). At the site of placenta detachment, the regenerating luminal epithelium was still discontinuous at PPD2, with morphologically immature, flat cells with low cytoplasmic volume (**Figures 2E, I**). By PPD4, the luminal epithelium was fully restored by a mature layer of columnar epithelial cells covering the mesometrial endometrial stroma (**Figures 2F, I**). Also, by PPD4, glands, a hallmark of the endometrium, were visible.

### 3.3 Increased cell turnover and reorganization of vascular and lymphatic networks

**Figure 3:**
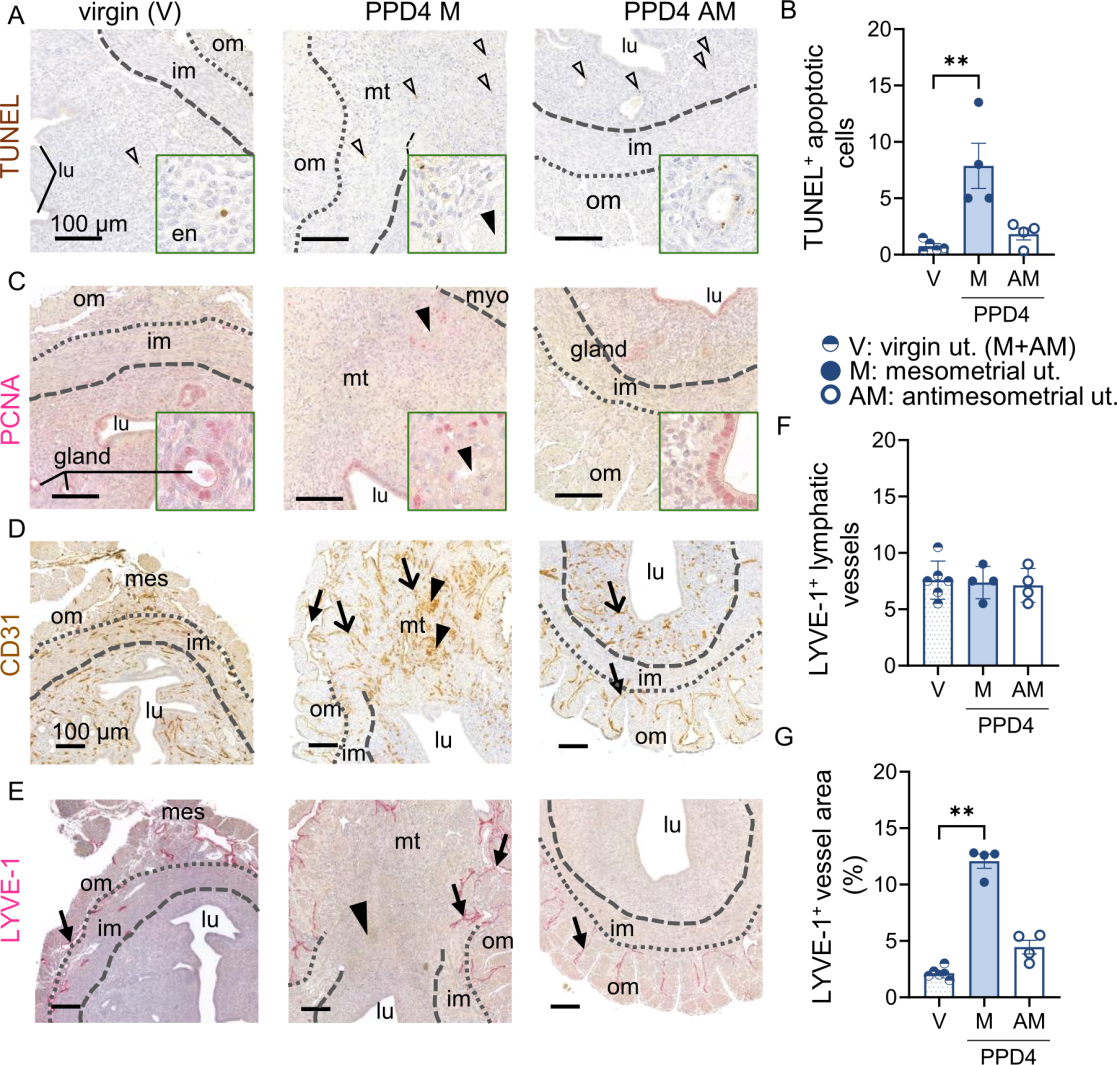
Increased apoptosis and discrete proliferation at the mesometrial uterus, rich in blood vessels and lymphatic drainage. **(A)** Representative photomicrographs of the distribution of apoptotic cells as evidenced by TUNEL+ (brown) in virgin (V) and PPD4 mesometrial **(M)** and antimesometrial (AM) uteri. **(B)** Quantification of TUNEL^+^ cells per visual field. **(C)** Representative images of PCNA positivity (pink) and **(D)** CD31 positivity (brown) from virgin and PPD4 uteri. **(E)** Representative images of uterine LYVE-1 positivity (pink) and **(F-G)** quantification of LYVE-1+ vessel count F and vessel area G per visual field. lu, lumen; im, inner myometrium; om, outer myometrium; dec, decidua (basalis); jz, junctional zone; mt, mesometrial triangle; sa, spiral artery; ac, amniotic cavity; myo, myometrium. Filled arrowheads: hemorrhagic clot, filled arrows: lymphatic vessels, empty arrowheads: DNA fragmentation (apoptotic cells), open arrows: blood vessels. Scale bars: 100 µm. In B, F, G mean with SEM, *p < 0.05, **p < 0.01; Kruskal-Wallis test.

We next analyzed the contribution of apoptosis and proliferation in the observed structural changes. Consistent with the profound mesometrial remodeling, TUNEL^+^ apoptotic cells were more prevalent in the mesometrial than in the antimesometrial PPD4 uterus (**Figures 3A, B**). Additionally, enhanced proliferative activity, particularly of PCNA^+^ cells around the mesometrial hemorrhagic clots (**Figure 3C**), was also observed. Proliferation also occurred in the glands and the luminal epithelium, which were gradually restored in the endometrium (**Figure 3C**). In contrast, cell death and proliferation were barely detectable in the myometrium (**Figures 3A, C**).

Furthermore, uterine vascular and lymphatic networks showed signs of intense remodeling in the postpartum status (**Figures 3D–G**). In PPD4, CD31^+^ blood vessels in mesometrial regenerating areas exhibited notable heterogeneity, with vessels of varying sizes (**Figure 3D**). In the vicinity of the clot, clusters of capillaries indicate incipient angiogenesis and a reorganization of the blood vessel network (**Figure 3D**). Lymphatic vessels were consistently found between the inner and outer myometrial layers, and, occasionally at the endometrial-myometrial interface (**Figure 3E**). While vessel numbers per area remained stable before and after pregnancy, relative lymphatic vessel area increased, particularly mesometrially, in PPD4 compared to virgin uteri (**Figures 3F, G**), suggesting enhanced lymphatic drainage of areas with most extensive postpartum remodeling including the restoration of the inner myometrial layer, resolving the hemorrhagic clots and mesometrial lymphoid aggregate of pregnancy.

### 3.4 Resorption of the mesometrial triangle is accompanied by reversion of myometrial dispersion

**Figure 4:**
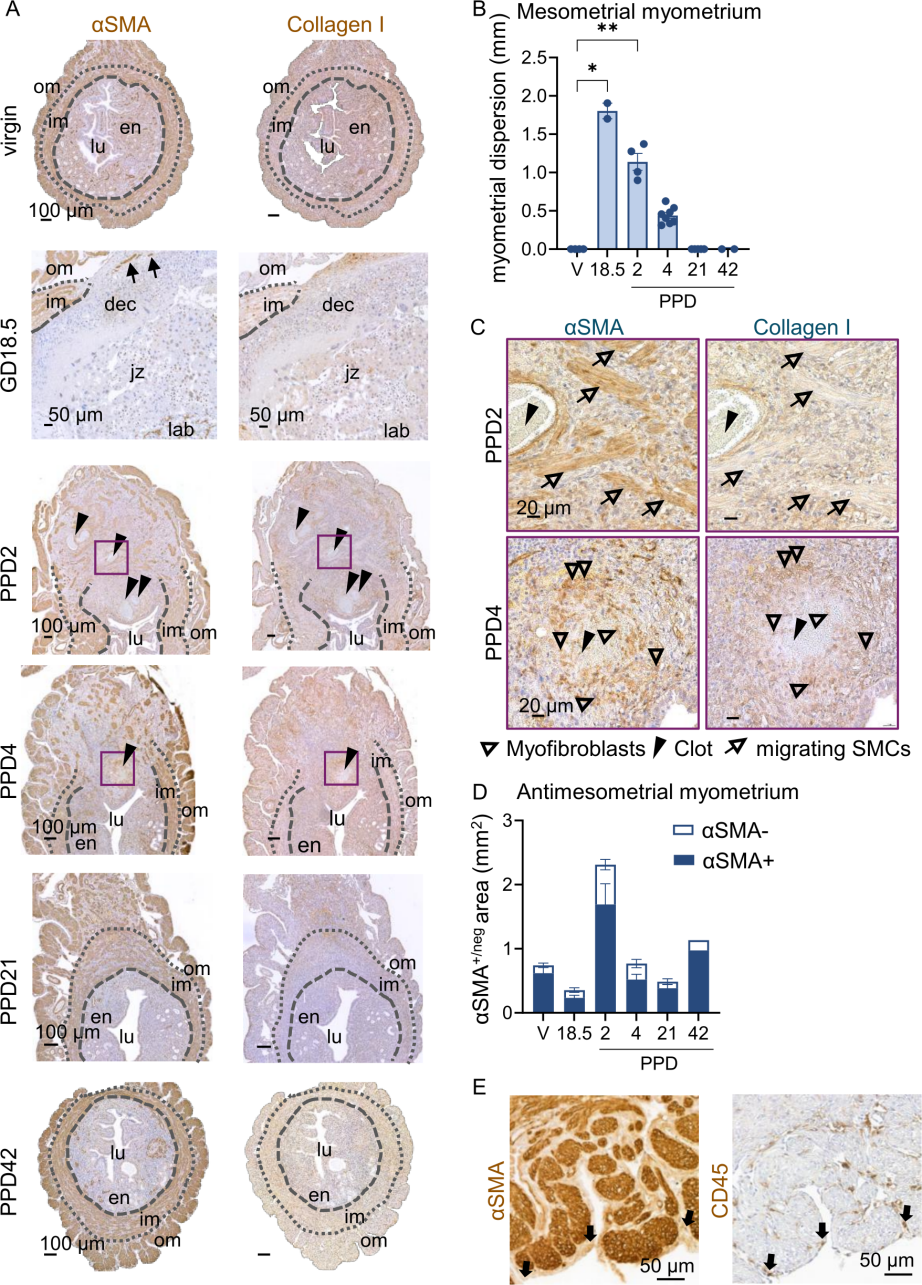
Postpartum mesometrial regeneration supported by myofibroblasts and CD45^+^ immune cells.**(A)** Representative photomicrographs of αSMA (left) and collagen I (right) immunohistochemical detection in virgin (V), GD18.5, PPD2, PPD4, PPD21, and PPD42 uteri. Filled arrowheads indicate hemorrhagic clot, filled arrows indicate smooth muscle bundles. **(B)** Evaluation of myometrial dispersion in uterine samples. **(C)** Representative photomicrographs of αSMA (left) and collagen I (right) immunohistochemical detection to show fibroblasts on PPD2 (top) and double positive myofibroblasts in a PPD4 uterus in the area around the clot (bottom). **(D)** Quantification of αSMA^+^ muscle fibers and αSMA^neg^ connective tissue area in antimesometrial region of uterine samples. **(E)** Representative photomicrographs of αSMA (left) and CD45 (right) immunohistochemical detection of PPD4 uterus in the outer myometrium (om). Full arrows indicate co-localisation of connective tissue (αSMA^neg^) and CD45^+^ cells. In **(B, D)** mean with SEM, *p < 0.05, **p < 0.01; Kruskal-Wallis test. im: inner myometrium, en: endometrium, lu: lumen, dec: decidua, jz: junctional zone, lab: labyrinth. Empty arrowheads: myofibroblasts, full arrowheads: hemorrhagic clot, empty arrows: migrating smooth muscle cells.

The downregulation of gene pathways related to muscle development and extracellular matrix (ECM) deposition in the human postpartum myometrium, together with the sharp reduction in mouse uterine size, despite minimal myometrial apoptosis, prompted us to investigate the postpartum myometrial involution in mice. Intriguingly, in late pregnancy (GD18.5), the inner myometrial layer was disrupted at the implantation site via myometrial dispersion (**Figures 4A, B**; **Supplementary Figure 2**), a less-described phenomenon stemming from uterine stretching (33). Paralleling the postpartum mesometrial triangle resorption, the distance between the posterior and anterior inner myometrial layers gradually closed, forming a continuum by PPD21 (**Figures 4A, B**). This likely resulted from a displacement of the existing myometrial fibers rather than hyperplasia, as cells were largely negative for Proliferating Cell Nuclear Antigen (PCNA). Intriguingly, abundant elongated αSMA^+^ collagen-I^neg^ cells, likely migrating smooth muscle cells undergoing differentiation into (myo)fibroblasts (34, 35), scattered through the mesometrial triangle on PPD2 (**Figure 4C**). By PPD4, activated αSMA^+^ and collagen I^+^ myofibroblasts, reportedly with a high contractile nature (36), were detected, e.g., around the mesometrial hemorrhagic clot on PPD4 (**Figure 4C****),** consistent with their involvement in wound healing (37).

Antimesometrially, a peak in αSMA^+^ fibers and αSMA^neg^ connective tissue transversal area on PPD2, supports a sustained contraction at this time (**Figures 4A, D**). By PPD4, however, αSMA^+^ fibers and connective tissue had recovered an area similar to that of virgin individuals. Of note, myometrial CD45^+^ immune cells, critically involved in the onset of labor (38) and with upregulation of activation pathways on human PPD7 myometrium, located exclusively in the connective tissue surrounding αSMA^+^ muscle fibers (**Figure 4E**).

### 3.5 Tissue resident macrophage subsets accumulated in the postpartum mesometrial uterus

**Figure 5:**
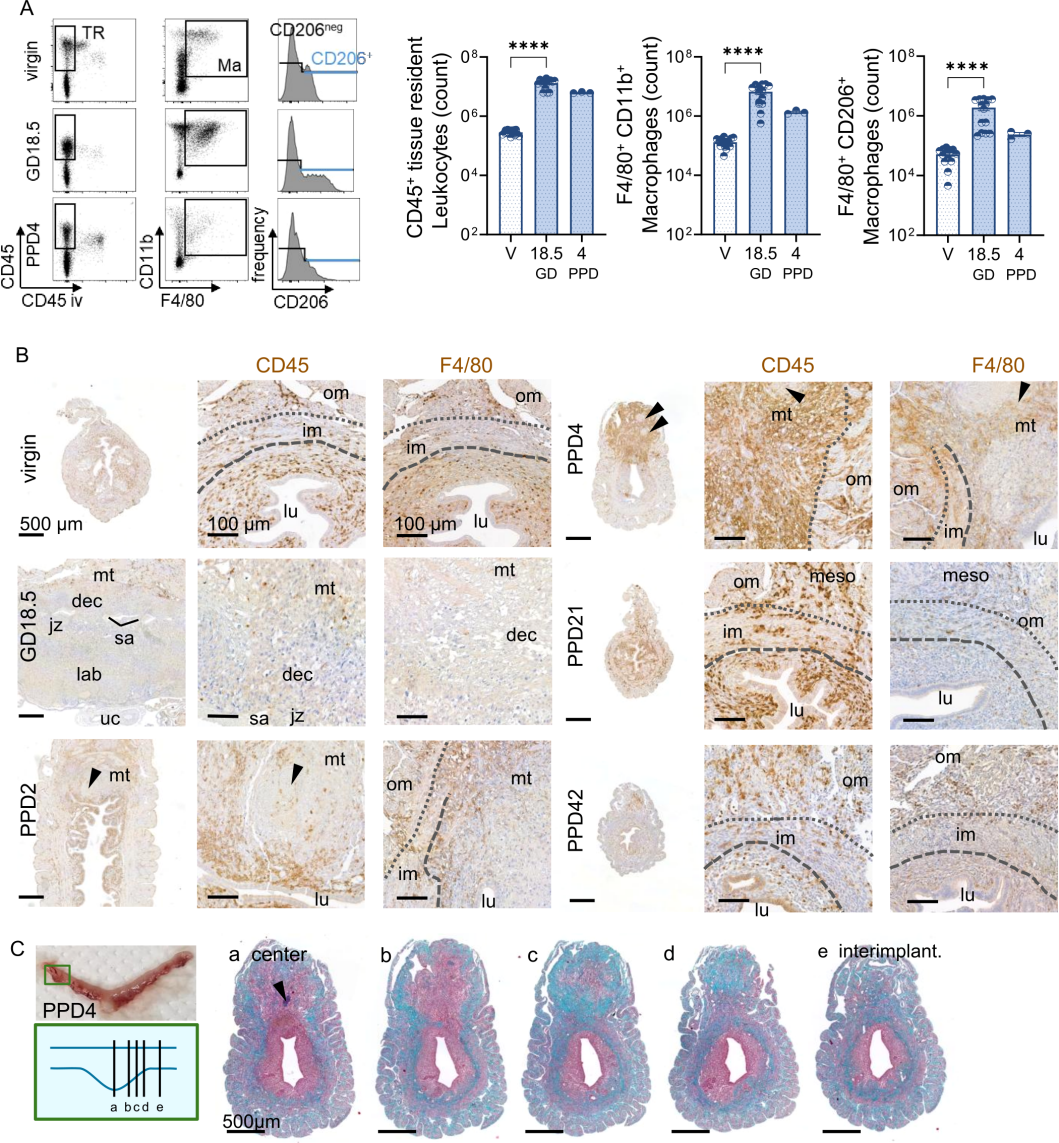
Accumulation of CD45^+^ leukocytes surrounded by a collagenous capsule early postpartum. **(A)** Analysis of flow cytometry data of virgin (V), GD18.5, and PPD4 uterus. Gating strategy, Histogram normalized to mode (left). Absolute number of CD45^+^ immune cells counts per tissue was calculated by multiplying the population of CD45^+^ cells with the mean of total cell count of the tissue suspension per group. Absolute number of macrophages was calculated by multiplying the population of F4/80^+^CD11b^+^ cells with CD45^+^ population multiplied by the mean of the total viable cell count per tissue of this group. The same calculation was applied to F4/80^+^ CD206^+^ population. Mean with SEM, *p < 0.05, **p < 0.01, ****p < 0.0001; Kruskal-Wallis test. TR, tissue resident; Ma, Macrophages; iv, intravenously. **(B)** Representative pictures of CD45 and F4/80 immunohistochemistry of virgin uterus; GD18.5. PPD2; PPD4; PPD21; and PPD42.lu, lumen; mt, mesometrial triangle; meso, mesometrium; im, inner myometrium; om, outer myometrium; dec, decidua (basalis); jz, junctional zone; sa, spiral artery; uc, umbilical cord. Filled arrowheads: hemorrhagic clot. **(C)** Visualization of a PPD4 murine uterus with one previous implantation site highlighted (green box). **(a-e)** Representative images of Masson Goldner staining of a PPD4 uterus taken in different depths for visualization of the collagenous capsule (blue) surrounding each implantation site.

Due to the steep upregulation of immune-related genes in scRNA-seq data and the upstream role of macrophages in tissue healing, we next evaluated F4/80^+^ macrophages (**Figures 5A, B**) in the peripartum uterus. Flow cytometry analysis revealed that tissue resident (negative for i.v. anti-CD45 antibody) (39) uterine CD45^+^ leukocytes, CD11b^+^ F4/80^+^ macrophages, many co-expressing CD206 receptor, increased during pregnancy and remained elevated on PPD4 (**Figure 5A**). Consistent with an alternatively activated-like profile (40, 41), CD206^+^ macrophages presented enriched expression of MerTK compared to CD206^neg^ macrophages (**Supplementary Figure 3**). Spatially, on PPD2-4, CD45^+^ and F4/80^+^ immune cells gathered in a clearly defined mesometrium region that coincided with the myometrial dispersion, detachment clots, and the former mesometrial lymphoid aggregate of pregnancy (**Figure 5B**). This leucocyte cluster scarcely spilled over the surrounding tissue and was in fact constraint within a 3-D capsule rich in collagen (**Figure 5C**, blue), among which collagen I was not a majoritarian component (**Supplementary Figure 1**). By PPD21, this inflamed area was largely resorbed and CD45^+^ cells distributed throughout the uterus, as were the less abundant F4/80^+^ macrophages (**Figure 5B**).

**Figure 6:**
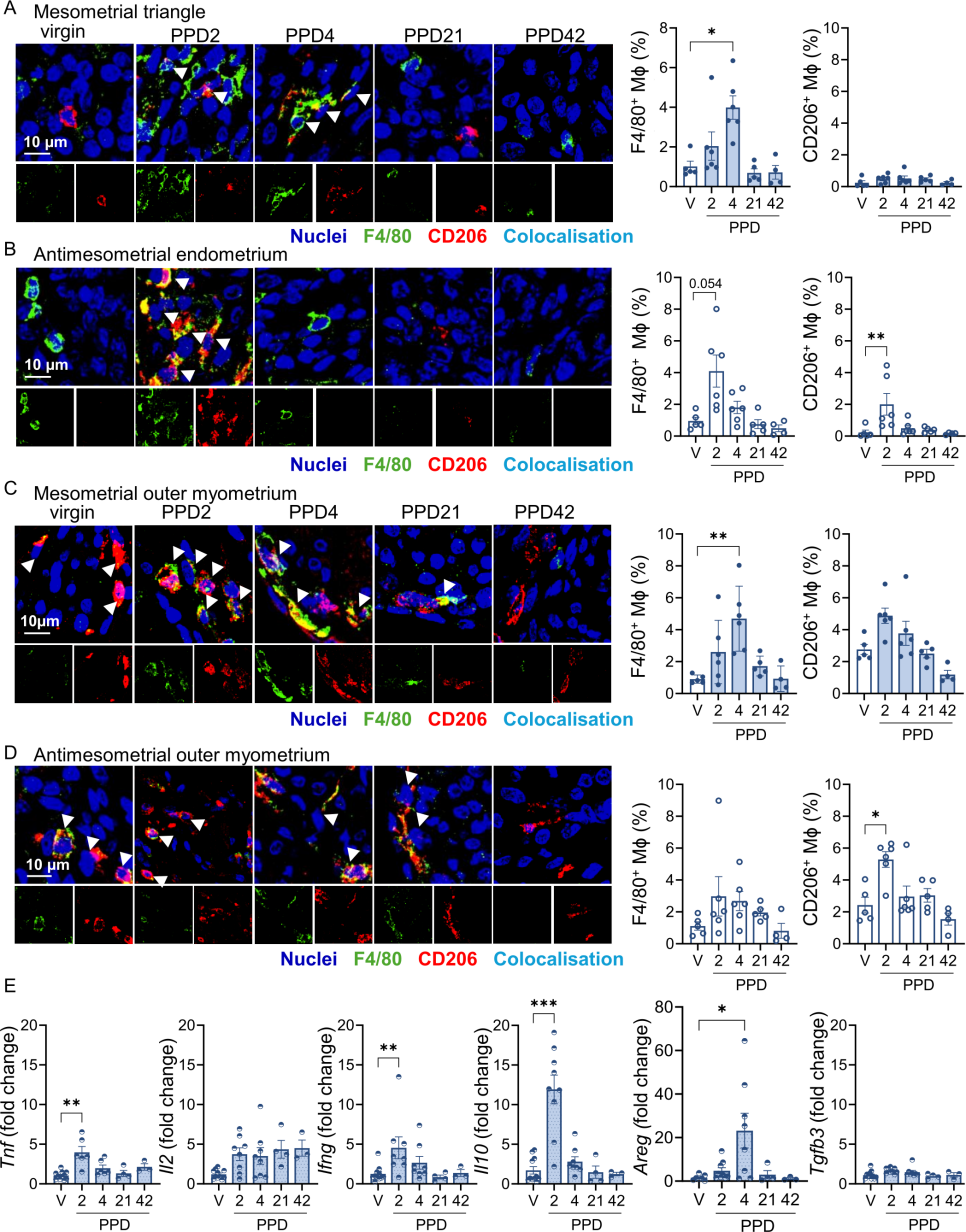
Uterine infiltration of F4/80^+^ macrophages in the myometrium and endometrium early postpartum. **(A-D)** Quantification of F4/80^+^ and CD206^+^ (right) macrophages with representative images of confocal microscopy (left) stained for F4/80 (green) and CD206 (red), in the mesometrial triangle **(A)**, antimesometrial endometrium **(B)**, mesometrial outer myometrium **(C)**, antimesometrial outer myometrium **(D)**, based on histological staining of uterine tissue sections from virgin, PPD2, PPD4, PPD21, and PPD42. White arrowheads show colocalization. **(E)** *TNF, Il2, IFng, Il10, AREG, Tgfb3* expression detected by qPCR in uterus. Ct values classified as ‘undetermined’ were excluded from the graphical analysis. A-E Mean with SEM, *p < 0.05, **p < 0.01; Kruskal-Wallis test.

In-depth spatial analyses revealed that largely CD206^neg^ macrophages, either inflammatory or non-polarized, accumulated in the mesometrial triangle by PPD4 (**Figure 6A**). Conversely, in the antimesometrial endometrium, macrophages, including CD206^+^ subsets, peaked by PPD2 and dropped thereafter (**Figure 6B**). In the myometrium, macrophages predominantly displayed a F4/80^+^ CD206^+^ alternatively activated-like profile, generally associated with tissue remodeling, and tended to increase peripartum, albeit only significantly in PPD2 antimesometrial myometrium, and gradually restored frequencies similar to those in virgin uteri (**Figures 6C, D**).

To further elucidate the immune milieu accompanying uterine remodeling, we analyzed cytokine levels within the entire uterus. mRNA levels for the inflammatory cytokines *Tnf* and *Ifng* were upregulated on PPD2, as were *Il2* levels, although this last non-significantly (**Figure 6E**). Even more pronouncedly, antiinflammatory *Il10* expression was also upregulated at this time point (**Figure 6E**). Simultaneously, mRNA for *Areg*, critical for restoration of tissue integrity (42), was increased on PPD4, whereas T*gbf3*, involved in scarless healing in the skin (43) remained unchanged (**Figure 6E**). This dichotomy of pro- and anti-inflammatory environment supports a balanced immune response that underlies uterine healing.

### 3.6 Newly recruited CD8^+^ T cells undergo differentiation in the postpartal uterus

**Figure 7:**
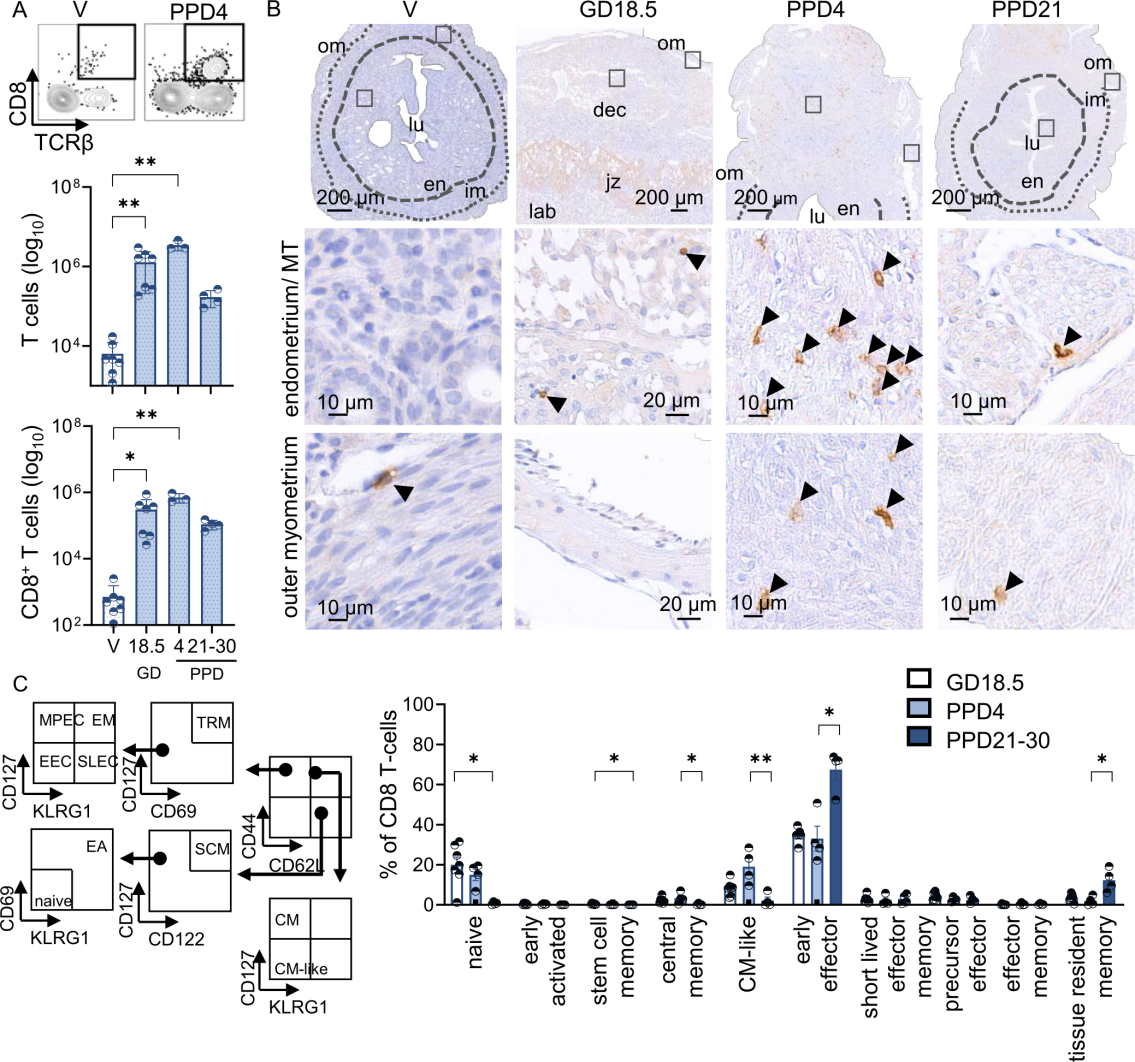
CD8^+^ T- cell characterization during late pregnancy and postpartum. **(A)** Analysis of flow cytometry data of virgin, GD18.5, PPD4 and PPD21–30 uterus **(B)** Representative photomicrographs of CD8 Immunohistochemistry of virgin uterus, GD18.5. PPD2, 4, 21-30. lu: lumen, mt: mesometrial triangle, meso: mesometrium, im: inner myometrium, om: outer myometrium, dec: decidua (basalis), jz: junctional zone, sa: spiral artery, en: endometrium. Filled arrowheads: CD8^+^ T-cells. **(C)** Diagram to show gating strategy (left) and quantification of CD8^+^ T cell subsets determined by flow cytometry (right). TRM, tissue resident memory; MPEC, memory precursor cells; EM, effector memory; SLEC, short-lived effector cells; EEC, Early effector cells; CM, central memory; SCM, stem cell memory; EA, early activated. **(A, C)** Mean with SEM, *p < 0.05, **p < 0.01, Kruskal-Wallis test.

Based on the pivotal role of uterine CD8^+^ T cells during pregnancy (9) and the scRNA-seq observations showing that T cell pathways were significantly affected in the postpartum period, we next analyzed these cell subsets. Flow cytometry analysis showed that tissue resident T cell and CD8^+^ T cell numbers significantly increased on PPD4 compared to the virgin uterus (**Figure 7A**) and remained higher even on PPD21-30. Indeed, CD8^+^ T cells abundantly seeded the mesometrial triangle on PPD4 and distributed after across the endometrium (**Figure 7B**). Generally, CD8^+^ T cells were more rarely detected in the myometrium (**Figure 7B**). Further phenotypical characterization revealed that in the postpartum phase, CD8^+^ T cells gained a more activated profile, with increased effector cells and tissue resident memory cells, expressing residency receptors and likely to be retained long term (**Figure 7C**).

## 4 Discussion

In the postpartum phase, the restoration of the endometrial epithelium and stroma is essential for maintaining fertility (44). Importantly, the myometrium also undergoes significant remodeling, including the gradual recovery of shape and size throughout the postpartum phase. As observed in the mouse, the myometrium, rich in CD206^+^ macrophages, appears to be safeguarded from mesometrial inflammatory foci, which are profusely surrounded by a collagen capsule. These findings highlight pronounced compartmentalization within the dynamic postpartum uterine remodeling needed for the uterus to regain homeostasis. The experimental investigation of the most affected pathways was guided by observations in scRNA-seq data from the PPD7 human uterus, underscoring pronounced innate and T cell activation, extracellular matrix deposition, modulation of the lymphatic endothelium, and myometrial regression. The finding of similar changes in the murine postpartum uterus validated its use as a suitable model for investigating these pathways. In fact, we further identified three distinct uterine compartments undergoing healing processes. They entail the regeneration of the endometrium, the resorption of the mesometrial triangle, and the restoration of the myometrium, which assemble synchronously to achieve full tissue recovery.

Consistent with its invasive and proliferative ability (45), we observed rapid endometrial regeneration following peripartal threading of the decidua. The upregulation of the mannose receptor CD206 and the proto-oncogene tyrosine-protein kinase MER, both of which can trigger phagocytosis in macrophages, indicates increased phagocytic activity, which is essential for macrophage polarization toward a tissue remodeling state (46–48). Conversely, in mice with surgically induced uterine adhesions, a predominance of CD301^+^ inflammatory macrophages shifted endometrial stromal cell differentiation toward αSMA^+^ myofibroblasts, which, through ECM secretion, promoted endometrial scar formation (49). These contrasting conditions show the critical role of macrophage phenotype and balance in coordinating reepithelialization, tissue remodeling, and the outcome of endometrial wound healing.

Compared to the endometrium, leukocyte and macrophage infiltration is most prominent at the mesometrial triangle. During pregnancy, this region (50) hosts a lymphoid aggregate rich in IFN-γ-producing uterine natural killer cells involved in vascular adaptation to pregnancy (51). In late gestation, the mesometrial triangle instead gathers type 2 innate lymphoid cells, dendritic cells, and IL33-expressing PDGFRα^+^ interstitial fibroblasts, which are involved in initiating labor (50). Postpartum, we observe that immune cells, including macrophages, rapidly and extensively infiltrate this tissue zone, engulfing hemorrhagic clots formed by placental detachment until they resolve. In this region, it has been shown that macrophages contribute to the clearance of decidual senescent cells during the postpartum period, and their depletion led to an accumulation of senescent cells (52). Notably, increased senescent cell burden has been associated with reduced fertility in a model of preterm birth in the same study, indicating an important role of macrophages in recovering the function of the uterus postpartum. We observed CD206^neg^ macrophages populating the mesometrial triangle, along with increased levels of *Tnf, Il2*, and *Ifng*, collectively supporting an inflammatory immune response in the early postpartum days. Notably, these cytokine levels were evaluated in whole uterine tissue. Thus, the observed changes may underestimate changes of some cytokines in inflamed zones, such as the mesometrial triangle, and overestimate them in less affected areas. Given the soluble nature of cytokines, future evaluation of the spatial distribution of active cytokine levels in the postpartum uterus is critically needed to gain insights into distinct, simultaneous responses across uterine compartments and their reciprocal influence.

Indeed, intratissue immune profiling and communication play critical roles in shaping cytokine responses. For instance, in the gut, immature CD206^neg^ macrophages produce low levels of IL-10, thereby supporting an inflammatory milieu (53). In contrast, in the hypoxic placental-detachment area, alternatively activated arginase-1^+^ macrophages with high phagocytic activity increased after cesarean section (14), supporting a role of labor in the polarization of macrophages at the wounds left by placental detachment. Based on our results and others, it remains elusive how macrophages support healing in this area. Macrophages can facilitate remodeling processes by secreting growth factors and chemotactic mediators and by promoting myofibroblast differentiation (54). Consistently, we detected activated αSMA^+^ collagen I^+^ myofibroblasts scattered throughout the mesometrial triangle and surrounding hemorrhagic clots, as these cells typically contribute to wound healing (36, 55). In injured intestinal mucosa, recruited macrophages can further aid, for example, by reprogramming epithelial stem cell proliferation and regeneration (56) and by forming a perivascular barrier that might prevent bacterial translocation (57). Further empirical work is required to determine whether these mechanisms also apply in the uterus, i.e., to protect the wounded site from ascending bacteria during parturition. Such a barrier could be further supported by the collagen-rich capsule that surrounds and isolates the wounded and inflamed site from the uterine cavity, since studies suggest that, similar to what is described for bacterial translocation (56), lymphocytes have limited migratory ability in dense ECM and collagen networks (58, 59).

Unlike the extensively examined role of macrophages in wound healing (41, 54, 60–62), the involvement of adaptive lymphocytes, e.g., CD8^+^ T cells, is less well defined. We observed that CD8^+^ T cells newly recruited to the mesometrial triangle continue to acquire effector and tissue-resident profiles until late stages of healing. Intriguingly, in addition to cytotoxic function, CD8^+^ T cells have been reported to promote vascularization at the feto-maternal interface (9) and to contribute to wound healing in humans by secreting amphiregulin (AREG). AREG, in turn, can act on the endothelium and pericytes, promoting myofibroblast differentiation, collagen production, and αSMA production to support wound healing (63, 64). Likewise, we observed elevated *Areg* expression in PPD4 uteri, although its cellular source remains unknown. As AREG can be produced by various immune and stromal components (63), further studies are required to unveil the mechanisms and players involved in AREG upregulation in postpartum uterine healing.

Taken together, the inflamed postpartum mesometrial triangle on PPD2–4 resembles, to some extent, granulation tissue around, e.g., a skin wound (55, 65) with accumulation of inflammatory macrophages and T cells, activated myofibroblasts, angiogenesis, and extracellular matrix deposition, to enhance tissue healing (66). As described in granulomas (67), this granulation-like tissue was surrounded by a collagen-rich extracellular matrix capsule, which may help contain and concentrate the inflammatory responses until resolution (68). In line with this, lymphatic vessels remain excluded from the mesometrial triangle within the myometrial compartment, albeit enlarged on the mesometrial side, highlighting the critical role of lymphatic drainage in maintaining tissue homeostasis. The accumulation of pro-inflammatory macrophages on PPD4 aligns with the inflammatory phase of skin healing, which may be transitioning to a proliferative phase, as indicated by the presence of granulation-like tissue (5).

The resorption of the mesometrial triangle, along with reduced uterine stretching, may be sufficient to reconnect the dispersed smooth muscle bundles, thereby restoring the inner myometrial layer by PPD21 rather than through muscle hyperplasia. Early postpartum, prominent myometrial contraction is evident as an increase in transverse tissue area. In mice undergoing cesarean section, such contractions help restore uterine size and promote healing of smooth muscle cells, blood vessels, and fibrin deposits (14). It remains unknown whether in human pregnancies the myometrium also disperses to accommodate the growing conceptus. Here, we took advantage of scRNA-seq data from a human postpartum uterus. The resulting data should be interpreted with caution, as the analysis, based on a single sample, may introduce individual biases compared to the population. Still, these results remain extremely valuable given the rarity and limited availability of such samples. Indeed, these exploratory insights into human postpartum wound healing set the stage for modeling these changes in mice. Importantly, the observations from human scRNA-seq closely align with the in-depth investigation of the process in mice, supporting both the validity of the individual human sample and the value of the mouse as a model of postpartum uterine physiological healing. In particular, the upregulation of leukocyte chemotaxis, myeloid leukocyte activation, and positive regulation of T cell activation in the human PPD7 uterus was reflected in the murine model by the recruitment of CD45+ leukocytes, including F4/80+ macrophages, T cells, and CD8+ T cells on PPD4. Hence, despite differences between species, conserved mechanisms are present in both human and mouse, including collagen synthesis, immune system, cell-cell adhesion, and extracellular matrix formation, as observed by us in the uterus, and by others in additional tissues (69, 70) which allows comparison of these mechanisms between the species.

Such muscle fiber involution may guide the restoration of shape and elastic properties of the uterus. Alternatively, activated-like CD206^+^ macrophages in the myometrial connective tissue may be actively involved in this process. In fact, growing evidence suggests that the myometrium actively influences labor induction and postpartum regeneration. Prenatally, mechanical stretching and hormonal signals stimulate chemokine expression, neutrophil and macrophage infiltration, thereby supporting inflammation and creating a feedback loop that activates myometrial cells to initiate labor (11). Elevated postpartum monocyte infiltration and increased inflammatory factors, together with enrichment of inflammatory macrophages, have been hypothesized to subsequently support myometrial involution in rodents (38). In line with these observations, our scRNA-seq analysis of human PPD7 myometrium revealed enhanced expression of genes related to myeloid leukocyte activation and leukocyte chemotaxis, e.g., CCR1 and CCR2, expressed on monocytes and macrophages. Binding of these receptors with their ligands promotes recruitment of myeloid cells (71), further supporting our observations that immune cell recruitment is not a temporary feature of labor but is upheld postpartum to support wound regeneration. However, whether macrophages functionally contribute to these processes has yet to be determined. It remains unknown whether smooth muscle cells, as shown in the intestine (34), also contribute to structural remodeling. In fact, under stretch-induced matrix metalloproteinase conditions, resting intestinal smooth muscle cells can shift to a migratory profile (35), with the capacity to proliferate or differentiate into fibroblasts, contributing to extracellular matrix deposition (35). In this context, it is plausible that smooth muscle cells from the inner myometrium migrate into the mesometrial triangle to support postpartum tissue regeneration. Our data suggest that postpartum uterine regeneration is driven by contraction-dependent myometrial involution and remodeling, in which macrophages play a critical role. The functional shift of smooth muscle cells may represent an additional mechanism contributing to regeneration.

This pronounced remodeling of the myometrium during postpartum healing raises the question about how these changes might influence long-term function. Clinical observations showed that human myometrium undergoes lasting adaptations during the first pregnancy that persist to the next pregnancy, as advanced cervical ripening in the first pregnancy leads to a shorter duration of labor in the second pregnancy (72). In addition, sonographic analyses have shown that optimized cesarean section operation techniques can reduce scar formation, highlighting the extent of external influence on pathological healing (73). Taken together, this work advances the understanding of a framework for the complex, dynamic immune and tissue changes involved in wound healing and uterine tissue remodeling after natural delivery. Comparatively, we observed a more intense inflammatory response at the site of placental detachment than in the myometrium. However, the quick remodeling of the unharmed myometrium and the return to homeostasis may be significantly limited by inflammation caused by a cesarean wound. Since the myometrium is the primary tissue involved in scar formation, we identified potential targets for further research in the context of cesarean section and cesarean scar disorder, including communication between smooth muscle and immune cells within a unique inflammatory and endocrine environment. Finally, we also describe coordinated steps of regeneration in the mesometrial triangle, which undergoes extensive inflammation, ultimately restoring a scarless endometrium at the former implantation site and guiding the reversal of myometrial dispersion, thereby evidencing the intricate interactions between tissue layers required to achieve homeostasis in the postpartum uterus.

## Data Availability

The data presented in the study are deposited in the NCBI Gene Expression Omnibus (GEO) repository under accession number GSE260658.
