# Supplementary material for: The postpartum uterus reveals compartment-specific remodeling processes with distinct immune signatures
Source: Front Immunol. 2026 Jun 10;17:1824792. doi: 10.3389/fimmu.2026.1824792 (PMC13291743; doi:10.3389/fimmu.2026.1824792)
Supplement: Supplementary file 1 [file Table1.docx]

Supplementary Material

# Supplementary Figures


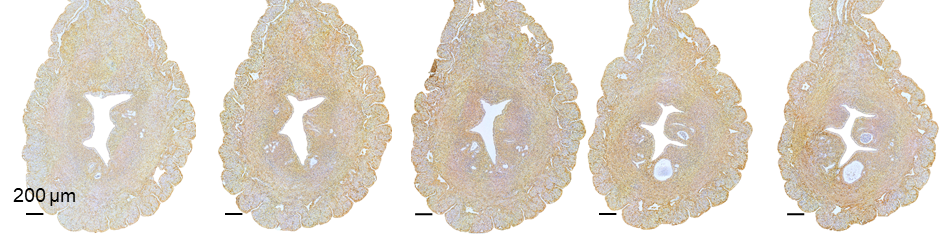


**Supplementary Figure 1.** Collagen I in different depths of the postpartum uterus


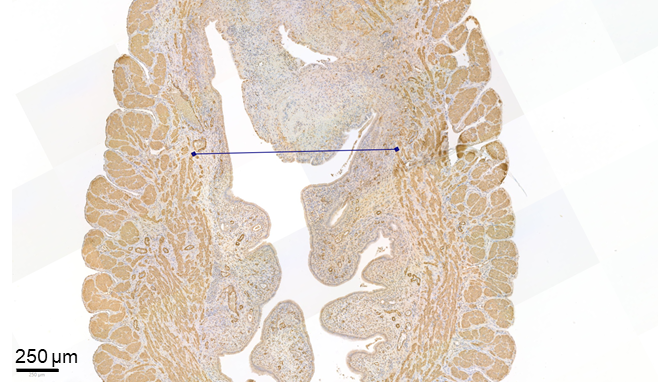
Representative images of a Collagen I histological stain in different depths of one former implantation site showing no major contribution to the collagenous capsule as shown by Masson Goldner Trichrome stain. Scalebar: 200 µm

Supplementary figure 2: Measurement of myometrial dispersion of inner myometrial layer

Representative visualization of the myometrial dispersion measurement in a PPD2 uterus stained for αSMA by immunohistochemistry. The distance between the extremes of the innermost myometrial layers on both sides were used as reference points for quantification of the degree of myometrial dispersion.


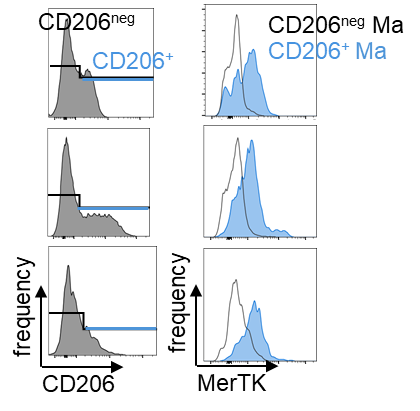


Supplementary Figure 3: Uterine CD206^+^ Macrophages are enriched in the expression of MerTK.

Flow cytometry analyses of F4/80^+^ CD11b^+^ CD206^+^ and CD206^-^ Macrophages, as depicted in figure 5 (left panel), evidenced enhanced expression of MerTK (right panel) in CD206^+^ (blue) when compared to CD206- (grey) macrophages. The histograms were normalized to mode.
